# Radial First: Paradox+Proficiency=Opportunity

**DOI:** 10.1161/JAHA.113.000281

**Published:** 2013-06-21

**Authors:** Sunil V. Rao, Mitchell W. Krucoff

**Affiliations:** 1 The Duke Clinical Research Institute, Durham, NC (S.V.R., M.W.K.)

**Keywords:** editorials, cardiovascular outcomes, percutaneous transluminal coronary angioplasty, transradial

## Introduction

*I would want the radial approach by an experienced person*.–Dr Robert M. Califf, MD*Vice Chancellor for Clinical and Translational Research, Director of the Duke Translational Medicine Institute, Professor of Medicine, Division of Cardiology, Duke University Medical Center, when asked about his own preferences should he ever need a percutaneous coronary intervention as told to Dr Adolph M. Hutter, MD, ACC Conversations with Experts, 2005*

## 

One of the lessons learned from the plethora of randomized clinical trials in cardiovascular medicine over the last 2 decades is the so‐called “quantitative interaction” of clinical therapeutics.^1^ This is the principle that the absolute benefit of a treatment is greater in patients at higher risk for the outcome. For example, acute coronary syndrome (ACS) patients with diabetes mellitus are at greater risk for adverse outcomes compared with nondiabetic patients. Therefore, a therapy that is shown to be efficacious in ACS likely will have greater absolute benefit in patients with diabetes. Incorporation of such a therapy into an ACS treatment pathway maximizes the likelihood that all eligible patients across the risk spectrum receive the therapy. In the absence of a uniform approach to treatment afforded by a pathway, treatment decisions are made on a case‐by‐case basis, increasing the likelihood that some patients are undertreated or not treated at all. Often, it is the patients at highest risk who are least likely to receive evidence‐based therapies. This “risk‐treatment” paradox has been described for a variety of different treatment strategies including the use of glycoprotein IIb/IIIa inhibitors and early invasive risk stratification for ACS,^2^ and the use of so‐called “bleeding avoidance strategies” for patients at high risk for bleeding.^3^

In this issue of the *Journal of the American Heart Association*, Wimmer and colleagues^4^ describe a risk‐treatment paradox for the use of radial access for percutaneous coronary intervention (PCI). Using data from 5 institutions in Massachusetts between 2008 and 2011, they analyzed over 17 000 patients who underwent PCI without requiring additional circulatory support to determine risk factors for access site complications. Patient characteristics like age, female sex, chronic kidney disease, peripheral arterial disease, diabetes, and prior PCI as well as procedure characteristics like emergent procedures were significantly associated with the occurrence of complications defined as access site bleeding requiring transfusion, large hematomas including retroperitoneal hemorrhage, vascular complications requiring imaging, or death from a vascular cause. Interestingly, for every 1% increase in the predicted risk of access site complications, there was a 14% to 17% lower likelihood of undergoing PCI with radial access. Since the radial approach is associated with a significant reduction in vascular complications compared with the femoral approach,^5^ and is now recommended by both the American^6^ and European guidelines,^7^ this study clearly demonstrates a risk‐treatment paradox. The reasons for this paradox are unclear, but some potential explanations for their findings are explored. For example, there may be an imbalance in the adoption of radial approach across operators and a patient‐level analysis may not account for some operators who routinely use radial access and others who do not. Indeed, the overall rate of radial approach in Massachusetts reported by the authors is much higher than previously published rates for the United States,^8^ suggesting that there are some high volume radial operators in the state. Another explanation, as acknowledged by the authors, is that their findings may be reflective of operators who are still early in their radial experience. The patients described as being high risk for access site complications in the study by Wimmer are also those in whom a radial approach can be more difficult or time‐consuming. It is entirely appropriate when learning the technique to “do no harm” and select patients who are less challenging. It would be very instructive to do a similar study in countries that have much higher rates of transradial procedures to see if the risk‐treatment paradox exists when a higher proportion of PCIs are performed via radial access. As operators go through the learning curve, transradial PCI in more complex patients and clinical settings, including patients with ST‐segment elevation MI, can be performed successfully. Therefore, it is important that the study by Wimmer not be taken as an indictment of appropriate patient selection among novice radial operators, but as an incentive to overcome the learning curve, become proficient, and then safely approach higher‐risk patients where the greatest benefit can be realized.

Unlike the risk‐treatment paradox for a medical treatment, which is often an error of omission, overcoming the selective use of a procedural technique is more challenging. It depends largely on an individual operator's proficiency with the technique and willingness to use it across the spectrum of patient risk. This is the equivalent of including an evidence‐based medicine on a treatment pathway—by making a procedural technique the default approach, the likelihood of a risk‐treatment paradox is much lower than if the decision is made on a case‐by‐case basis. Ultimately all patients are at risk for vascular complications, it is the gradient of risk that influences the magnitude of benefit from radial access. Moreover, there are other benefits to transradial PCI like early ambulation and patient preference^9^ that should be taken into account. Importantly, like any other medical procedure, a higher volume of radial procedures is associated with a higher success rate; however, this volume–outcome relationship extends beyond procedural metrics like successful radial arterial access and shorter procedure time. The available data show that there is a relationship between increasing proficiency with transradial PCI and the magnitude of benefit conferred to patients. This applies not only to safety parameters such as radiation exposure, but also to the “efficacy” of radial approach, that is, its effect on bleeding and vascular complications and perhaps even other outcomes like mortality.

At the most basic level, adoption of the radial approach is simply a change in the arterial access site from the femoral artery to the radial artery. However, since the radial artery is smaller in caliber than the femoral artery, and the route from the wrist to the heart crosses several other arterial vessels, challenges faced by the novice radial operator include obtaining arterial access, traversing a potentially tortuous radial or brachial artery (including the potential for arterial loops), entering the ascending aorta, and seating diagnostic or guiding catheters. All of these issues can prolong procedure time and increase the risk of procedure failure.^10^ As experience with radial procedures increases, the risks of access failure, overall procedural failure (defined as needing to change to femoral access), and even procedure time significantly decrease (Figure – Panel A).^11^ What is most revealing is that this “learning curve” is true for even experienced operators. The more radial procedures an operator performs, the better they get, even if they are already a radial “expert” (Figure – Panel B).^12^ Another issue that is of concern is the increase in radiation exposure associated with transradial procedures that has been documented in almost all studies comparing radial and femoral approaches. Again, more detailed analyses demonstrate that these differences are driven almost entirely by the learning curve. Among operators and centers experienced with transradial procedures, there is no significant difference in radiation exposure between procedures performed via radial or femoral access (Figure – Panel C).^13–14^

**Figure 1.**
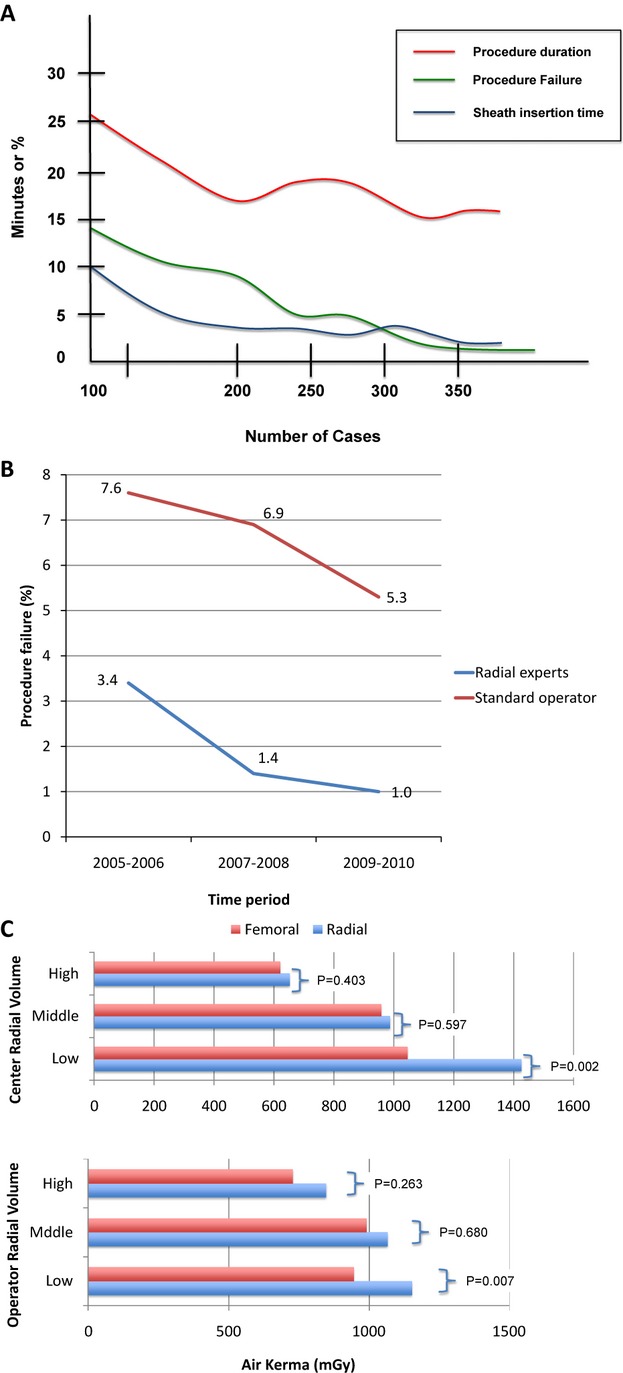
A, Relationship between radial experience and procedure duration, procedure failure, and sheath insertion time (adapted from Spaulding et al^11^). B, Relationship between radial experience and procedure failure among radial experts and standard operators (adapted from Burzotta et al^12^). C, Relationship between radial volume measured at the center level and operator level and radiation exposure measured as Air Kerma (mGy) (adapted from Jolly et al^14^).

Not only are procedural metrics improved with increasing experience, but clinical outcomes are also affected. The RIVAL (Radial Versus Femoral Access for Coronary Intervention) Trial was the largest randomized trial comparing radial with femoral access in over 7000 patients with ACS undergoing coronary angiography or PCI.^9^ Overall, there was no difference between the 2 arms with respect to the 30‐day occurrence of death, MI, stroke, or major bleeding. This outcome was surprising given the results of prior studies clearly showing an advantage of the radial over the femoral approach. Delving deeper into the RIVAL trial reveals 3 important findings: First, the bleeding events in the trial were predominantly unrelated to the access site; there was a benefit of radial access in reducing major vascular complications, which is an outcome highly sensitive to the randomized intervention. Second, the benefit of radial approach with respect to death, MI, or stroke was demonstrated in centers that had the most experience with radial procedures. Third, there was an association between radial access and reduced mortality among patients with ST‐segment elevation MI, a subgroup of patients who were most likely enrolled at centers with the highest radial proficiency. The first finding reflects the underlying rationale for the study by Wimmer and colleagues. The second and third findings again relate to the volume‐outcome relationship—the superiority of radial approach with respect to clinical outcomes is only realized when the operator and center are proficient with transradial procedures. In less experienced hands, there does not appear to be a difference in outcomes between radial and femoral access, with radial procedures having longer procedure times, more radiation exposure, and higher rates of bailing out to femoral access (ie, radial procedure failure).

These data taken in context with the results of the study by Wimmer provide an important path forward to improving the outcomes of patients undergoing PCI. The learning curve for radial procedures is relatively shallow (≈50 procedures according to one study^15^), and the process of becoming proficient consists of creating a “radial first” program. Such a program requires participation from the cardiologists, as well as the catheterization laboratory staff. Cardiologists should undergo formal radial training either by attending a course, being proctored by a radial expert, or both. The catheterization laboratory nursing and technologist staff should be trained in patient preparation, be made aware of what equipment is needed, and familiarized with the postprocedure recovery process. In addition, the catheterization laboratory team must have a problem‐solving attitude during the early radial experience to deal with procedural challenges and overcome the learning curve. Once the operator and catheterization laboratory staff have been educated on the basic technical aspects of radial procedures, and have experience with lower‐risk patients, there should be a commitment to use radial access in all eligible patients undergoing cardiac catheterization. Leveraging the expertise of local, regional, national, and international experts is an integral part of this process, and there are now multiple online resources for the novice radial operator to discover technical tips and tricks to facilitate transradial procedures.

Since improving patient outcomes depends on the application of evidence‐based treatment strategies, addressing the risk‐treatment paradox is an important challenge facing clinicians. The patients at highest risk for access site bleeding and vascular complications after PCI are those who stand to gain the most from the radial approach. Once the learning curve for transradial PCI is overcome, developing a “radial first” approach can minimize or even eliminate the risk‐treatment paradox. Given the amount of evidence supporting the benefits of radial access, interventional cardiologists should embrace the radial approach, do it often, become proficient at it, and use it as an opportunity to obtain the best outcomes.
